# A 12-Port MIMO Antenna System for 5G/WLAN Applications

**DOI:** 10.3390/mi14061196

**Published:** 2023-06-05

**Authors:** Wenshi You, Zhonggen Wang, Wenyan Nie, Weidong Mu

**Affiliations:** 1 School of Electrical and Information Engineering, Anhui University of Science and Technology, Huainan 232001, China; yws18815549204@163.com (W.Y.); zgwang@ahu.edu.cn (Z.W.); mwd18755802702@163.com (W.M.); 2 School of Mechanical and Electrical Engineering, Huainan Normal University, Huainan 232001, China

**Keywords:** 5G, slot antenna, MIMO antenna, WLAN, mobile communication

## Abstract

In this paper, a 12-port MIMO antenna system for 5G/WLAN applications is proposed. The proposed antenna system consists of two types of antenna modules: an L-shaped antenna module covering the C-band (3.4–3.6 GHz) for 5G mobile applications and a folded monopole module for the 5G/WLAN mobile application band (4.5–5.9 GHz). Each two antennas form a pair, six pairs in total, forming a 12 × 12 MIMO antenna array, and the elements between the antenna pairs can achieve an isolation of 11 dB or more without additional decoupling structures. Experimental results show that the antenna can cover the 3.3–3.6 GHz and 4.5–5.9 GHz bands with an overall efficiency greater than 75% and an envelope correlation coefficient less than 0.04. Finally, the one-hand holding mode and two-hand holding mode are discussed to demonstrate their stability in practical applications, and the results show that they still exhibit good radiation and MIMO performance when operating in both modes.

## 1. Introduction

The fifth generation of mobile communication (5G) has been officially put into commercial use, and one of its main features is the high-capacity and high-rate transmission of information [1]. Multiple-input multiple-output (MIMO) technology can effectively increase channel capacity and spectral efficiency and is a promising approach to meeting the demand for large data rates.

Currently, different countries and operators use different frequency bands for 5G communication. For example, LTE band 42 (3.4–3.6 GHz) and LTE band 43 (3.6–3.8 GHz) have been certified and adopted by the European Union [2]; Japan has licensed 5G bands from 3.6 to 4.2 GHz and 4.2 to 4.9 GHz [3]; and the 5170 to 5835 MHz band is classified as a 5G WLAN (wireless local area network) operating band [4].

However, for MIMO array antennas applied to cell phones, mutual coupling between MIMO units will be unavoidable due to the limited space of cell phones. Severe mutual coupling will lead to the degradation of isolation and seriously affect the channel capacity of MIMO systems [5]. Since the 3.5 GHz band (3.4–3.6 GHz) and the 5 GHz band (4.8–5 GHz) have been identified for 5G mobile communications, it is necessary to develop a MIMO antenna system that is suitable for 5G communication bands with high isolation at the same time.

To meet the needs of mobile communications, there is a large body of literature on designing MIMO antennas for 5G smartphone applications [6,7,8,9,10,11,12,13,14,15,16,17,18,19,20,21,22,23,24,25,26,27]. These systems have shown advantages in terms of isolation, envelope correlation coefficient (ECC) and antenna efficiency, while different techniques and decoupling structures have been proposed and investigated to improve the isolation, such as orthogonal mode isolation in [6], parasitic structure and polarization diversity in [7] and the neutralization line technique in [8,9,10,11,12,13,14]. The literature [15] proposes an integrated MIMO antenna with a coupled-loop structure based on the pattern cancellation method, which achieves self-decoupling. In [16,17,18,19], high isolation is achieved by loading antenna pairs decoupled by aggregate elements. Moreover, recently, a new dual-antenna MIMO structure based on spatial multiplexing, called an antenna pair [20,21,22,23], has been investigated. To decouple the antenna pairs, the vast majority of the antennas exploit resonant mode current cancellation in a shared radiator [24,25,26], while some of the antennas exploit the polarization orthogonality of the two antennas [7,27].

Based on the above status, this paper proposes a 12-port highly isolated broadband MIMO antenna system for 5G/WLAN applications. In order to support multiple communication modes and improve channel capacity, the proposed antenna system is made up of two different types of antenna modules. One is an L-shaped antenna, consisting of an L-shaped metal patch and a T-shaped slot, which covers the C-band (3.4–3.6 GHz) for 5G mobile applications. The second module is a folded monopole consisting of a meandering metal patch and a rectangular slot, and each antenna unit of this module covers 4.5–5.9 GHz used for 5G and WLAN mobile applications. Simulations (obtained by HFSS) and observations corroborate the performance of the proposed 12-port MIMO system.

## 2. Design of Proposed MIMO Antenna

**Figure 1:**
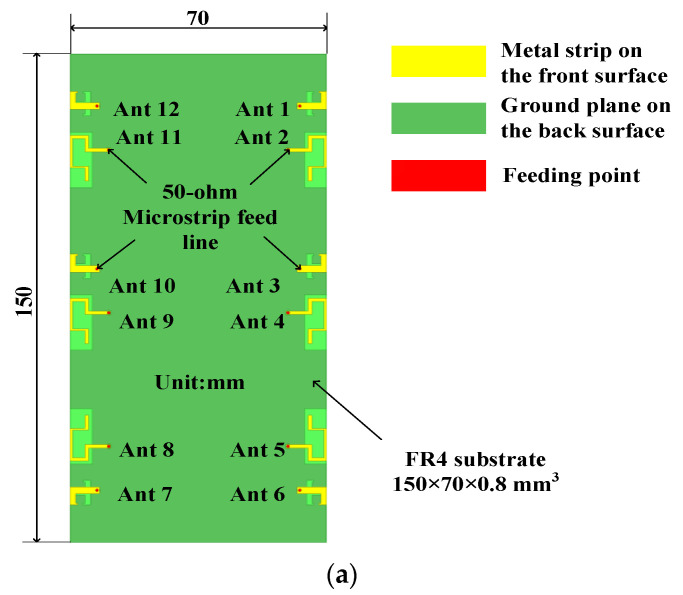

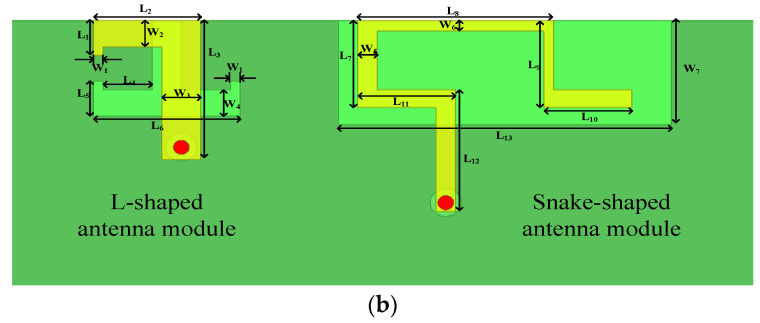
Geometry of the proposed MIMO antenna system: (**a**) Perspective view. (**b**) Detailed structure of the slot antenna element (taking Ant1 and Ant2 as an example).

The proposed 12-port antenna structure is shown in Figure 1. The antenna system consists of two types of antenna modules: an L-shaped antenna module consisting of a T-shaped slot and an L-shaped metal patch for 5G mobile applications in the C-band (3.4–3.6 GHz), and a folded monopole module consisting of a rectangular slot and a folded monopole patch for 5G/WLAN mobile applications in the frequency band (4.5–5.9 GHz). As shown in Figure 1a, there are six pairs of antenna units symmetrically distributed along the long side of the FR4 dielectric substrate (with a relative tolerance of 4.4 and a loss tangent of 0.02), and each antenna pair includes an L-shaped antenna and a folded monopole antenna, both of which are 8mm apart, forming a 12 × 12 MIMO antenna array. The standard system substrate size is 150 × 70 × 0.8 mm^3^, which is suitable for 5.5-inch smartphones. The metal ground (140 × 70 mm^2^) is printed on the back side of the FR4 substrate. The precise construction of the antenna components (Ant1 and Ant2 as examples) is depicted in Figure 1b. Table 1 shows the dimensions of the antenna elements.

### 2.1. L-Shaped Antenna Module

Each antenna element in the L-shaped antenna module is supplied via a 50-ohm L-shaped microstrip feed line linked to the ground plane via an SMA connection. On the bottom of the substrate, a T-shaped slot radiator is etched where the printed metal floor corresponds to the L-shaped antenna. This antenna, unlike traditional closed-slit antennas, employs an etched T-shaped slit radiator with open branches. T-slits boost antenna capacitance by inserting I-shaped opening branches into the center of the U-slit. The overall size of each element of the L-shaped antenna is 3 × 8 mm^2^, as shown in Figure 1b, and each antenna element covers the C-band of 5G mobile applications, i.e., 3.3–3.6 GHz.

### 2.2. Folded Monopole Module

In the folded monopole module, each antenna element consists of a rectangular ground-plane slot and a meandering metal strip. The folded monopole is printed on the FR4 substrate’s top layer. Each folded monopole has six 50 vertical feed strips and six horizontal feed strips for tuning. The antenna element may be set to the required frequency range by altering the width and length of the feed strips. The entire size of the folded monopole element encompassing the high-frequency range for 5G mobile applications and the WLAN band (4.5–5.9 GHz), as shown in Figure 1b, is 10 × 13.5 mm^2^.

### 2.3. Current Distribution

**Figure 2:**
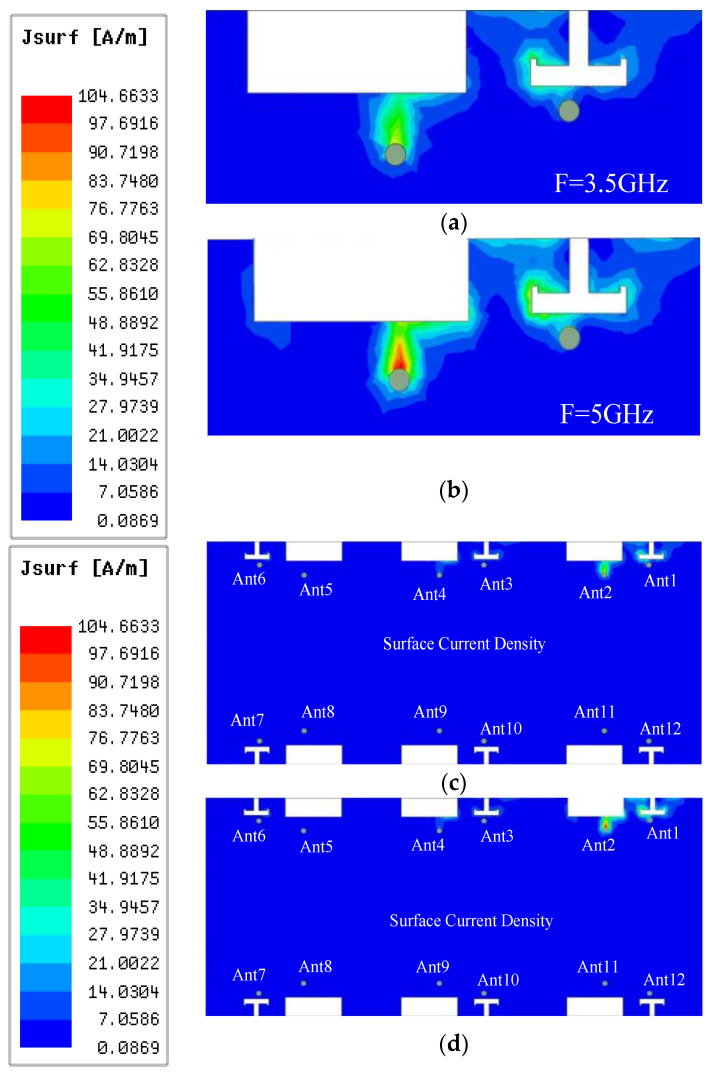
Surface currents on the antenna ground plane: (**a**) Local current distribution at 3.5 GHz. (**b**) Local current distribution at 5 GHz. (**c**) Overall current distribution at 3.5 GHz. (**d**) Overall current distribution at 5 GHz.

In order to study the working mechanism of the proposed antenna model, a parametric study of the individual antenna elements of the two modules was carried out using the electromagnetic simulator HFSS. Since the six antenna elements in each module have the same geometry, the parameters of Ant1 and Ant2 are used to analyze the excitation of the proposed antenna system in the low- and high-frequency bands. Figure 2 shows the current distribution in the ground plane of the antenna, which is convenient for a visual analysis of the mutual coupling effect between antennas in different resonant modes. As shown in Figure 2a, Ant1 shows a half-wave distribution in the resonant mode at the low-frequency band of 3.5 GHz, and its strong current with a length of 4 mm is mainly concentrated in the half of the T-slot, which indicates that the antenna works in the resonant mode of 0.38λ at 3.5 GHz (λ corresponds to the wavelength of 3.5 GHz). Similarly, Figure 2b shows the current distribution of Ant2 at 5 GHz excitation in the high-frequency band, where the surface current at 5 GHz is concentrated on one side of the rectangular slot with a length of about 7 mm, which is about 1.12λ of the corresponding wavelength at 5 GHz. Because the isolation between separate antennas is strong in Figure 2c,d, open decoupling is a significant strategy for reducing the coupling between antenna parts.

## 3. Results and Discussion

**Figure 3:**
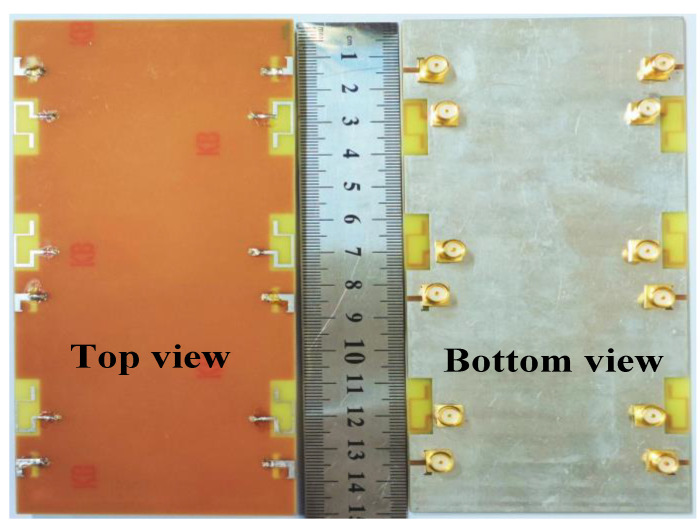
Fabricated prototype of the proposed 12-port MIMO antenna system.

**Figure 4:**
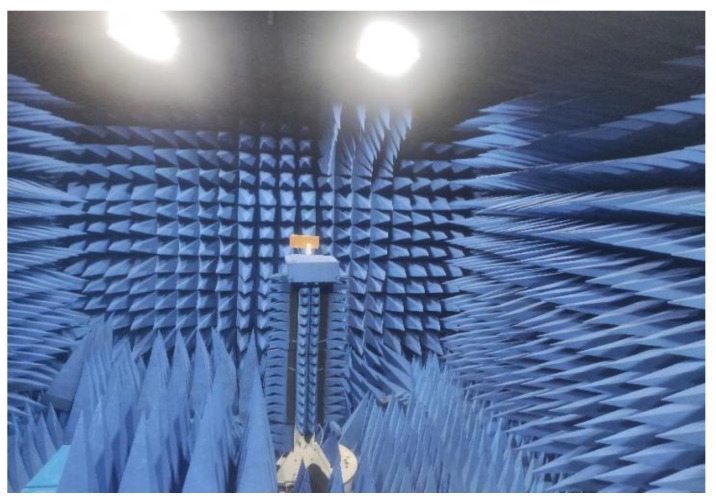
Testing environment of the proposed antenna in an anechoic chamber.

To verify the feasibility of the proposed antenna system, we fabricated and tested the proposed antenna model. Figure 3 depicts a 12-port MIMO antenna system. Figure 4 shows the antenna measurement environment anechoic chamber.

### 3.1. S-Parameter

**Figure 5:**
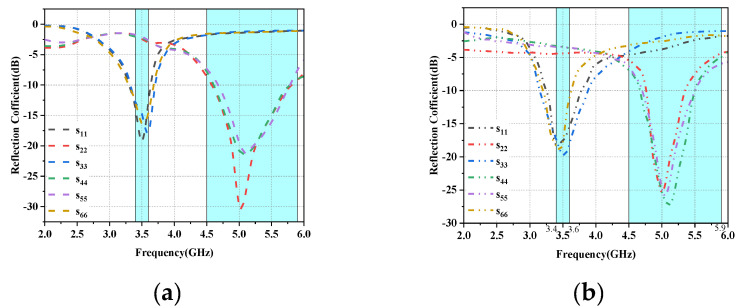
Reflection coefficient values and frequency for Ant1 to Ant6: (**a**) Simulated values. (**b**) Measured values.

**Figure 6:**
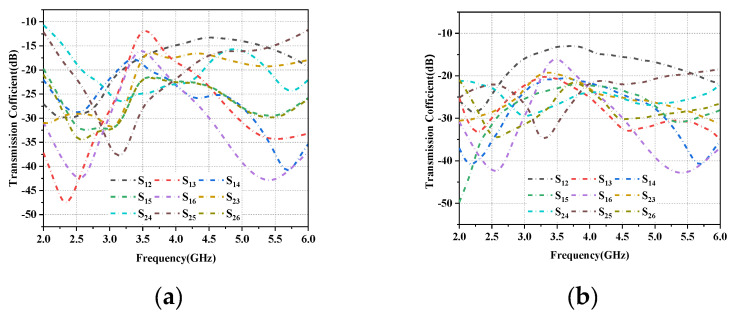
Coupling between different antenna pairs: (**a**) Simulated values. (**b**) Measured values.

Figure 5a shows the simulated reflection coefficient of each antenna unit versus antenna frequency, and it can be seen that the reflection coefficient in the operating band of the L-shaped antenna unit is −10 dB, while the reflection coefficient in the operating band required for the folded monopole unit is greater than −6 dB. Figure 5b shows the corresponding measured reflection coefficient values. Because the antenna units are distributed symmetrically on the left and right sides of the substrate, the reflection and transmission characteristics are the same, so Figure 4 and Figure 5 only show the results for the left side of the array (Ant1 to Ant6). The simulated and measured values for each element combination are shown in Figure 6a,b, respectively, and it can be seen that the isolation amplitude of each cell is greater than 11 dB in the desired 5G mobile application band and WLAN band. The small error between simulation and measurement may be influenced by the soldering and testing environment of the physical object.

### 3.2. Parametric Studies

The proposed array antenna design was investigated in depth by the simulation software HFSS version 20. To understand how the two-antenna module tuning mechanisms affect the impedance matching and operating bandwidth of individual antenna array elements, a parametric study was conducted with Ant1 and Ant2 as examples. It is worth noting that when one of the parameters is adjusted, the other parameters will remain unchanged.

**Figure 7:**
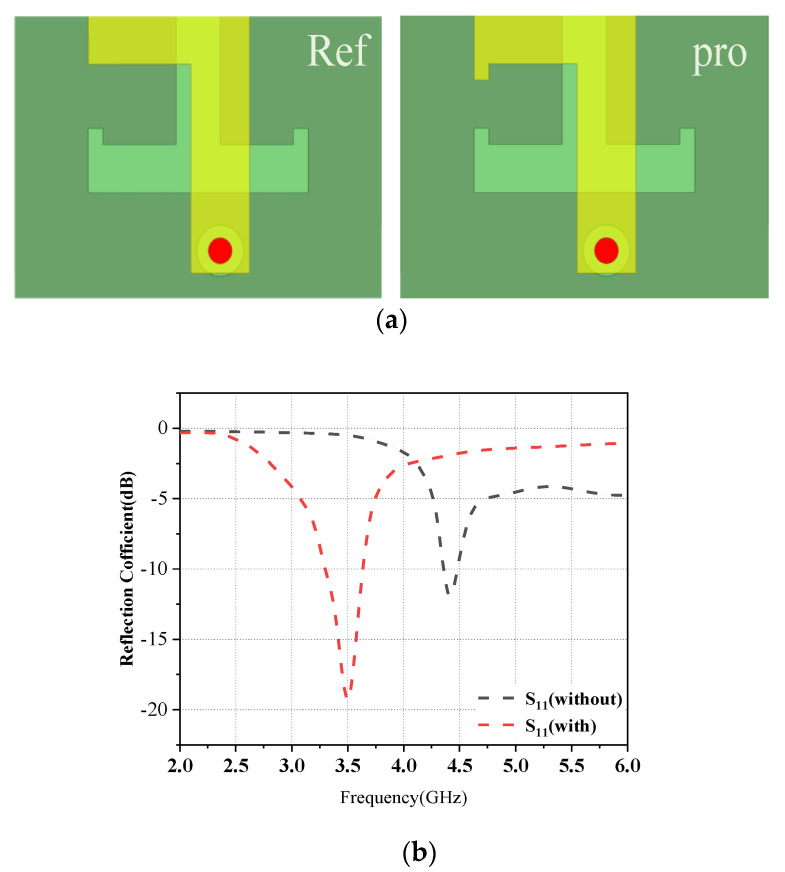
Design evolution of the antenna element structure: (**a**) The structure of the reference and proposed antenna. (**b**) The comparison of the reflection coefficient between the reference and proposed antenna elements.

In the L-shaped antenna module, unlike the conventional L-shaped metal patch, the proposed patch structure in this paper adds a small metal patch of 0.5 × 0.5 mm^2^ at the front of the horizontal microstrip line. Figure 7a compares the conventional L-shaped metal patch with the proposed metal patch, and Figure 7b shows the reflection coefficients of both. From Figure 7b, it can be seen that Ant1 operates in the frequency band of 4.2–4.6 GHz when using the conventional L-shaped metal patch, and by adding a small metal patch to the conventional L-shaped metal patch, the center frequency of Ant1d shifts to 3.5 GHz and shows superior impedance-matching characteristics.

**Figure 8:**
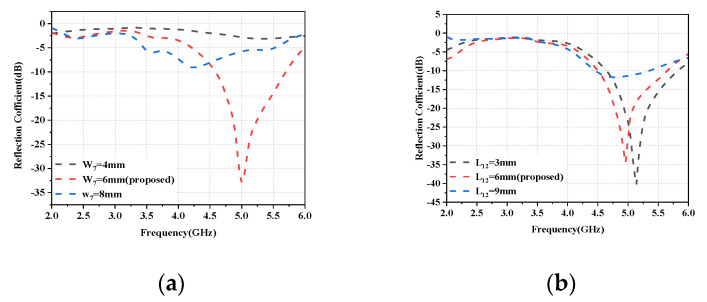
The simulated reflection coefficients of the folded monopole module as a function of different lengths and widths: (**a**) W_7_. (**b**) L_12_.

A parametric study was performed in the folded monopole module to investigate and analyze the effect of different lengths and widths, including W_7_ and L_12_. Figure 8a depicts the influence of W_7_ on the reflection coefficient by expanding the slot width from 2 mm to 8 mm, resulting in a resonant mode at 5.0 GHz. The slot width was lowered for higher frequency bands by reducing the width of W_7_, which narrowed the slot width and shortened the current flow. In summary, W_7_ may be used to adjust the antenna element’s center frequency in the appropriate frequency band. Figure 8b demonstrates that L_12_, another parameter that influences the resonance frequency, is altered from 3 mm to 9 mm. The resonant frequency is tuned to a higher band and the operating frequency is shifted to a higher frequency. Based on the above results, the W_7_ width is set to 6 mm and the L_12_ length is set to 12 mm, and the frequency band can be adjusted to the desired value.

### 3.3. Envelope Correlation Coefficient (ECC)

ECC is one of the main indexes to evaluate the performance of the MIMO antenna array. The concept of ECC is mainly used to quantitatively describe the mutual independence of the transmitted signals between different antenna units in the MIMO antenna array: the smaller the value of ECC, the greater the mutual independence of the transmitted signals between different antenna units in the MIMO antenna array. In MIMO antenna design, the value of ECC is required to be less than 0.5 [28]. The most common method to calculate ECC is to use the S-parameter of the MIMO antenna array, and the formula is as follows [29]:

(1) $$\mathrm{ECC}=\frac{{\left|\iint_{4\pi }\left[E_{1}\left(\theta ,f\right)*E_{2}\left(\theta ,f\right)\right]d\Omega \right|}^{2}}{\iint_{4\pi }{\left|E_{1}\left(\theta ,f\right)\right|}^{2}d\Omega \iint_{4\pi }{\left|E_{2}\left(\theta ,f\right)\right|}^{2}d\Omega }$$

**Figure 9:**
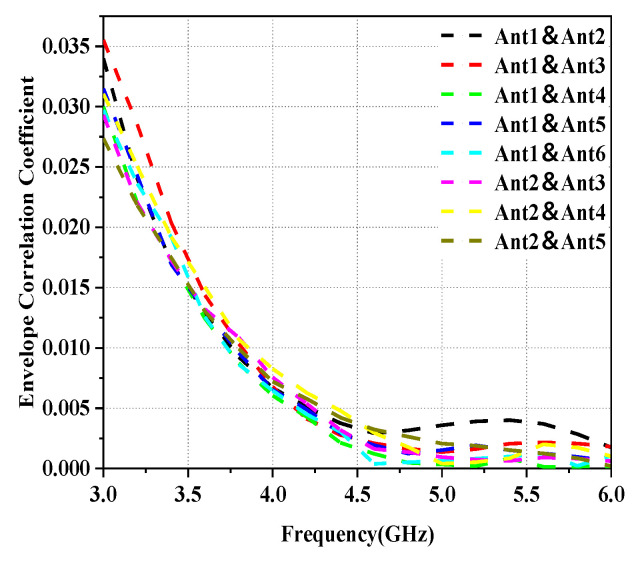
The calculated envelope correlation coefficient of the proposed MIMO antenna system.

As can be seen in Figure 9, the ECC value calculated for the proposed design is less than 0.04 over the entire operating frequency range, thus indicating a good diversity performance.

### 3.4. Radiation Efficiency and Antenna Peak Gain

**Figure 10:**
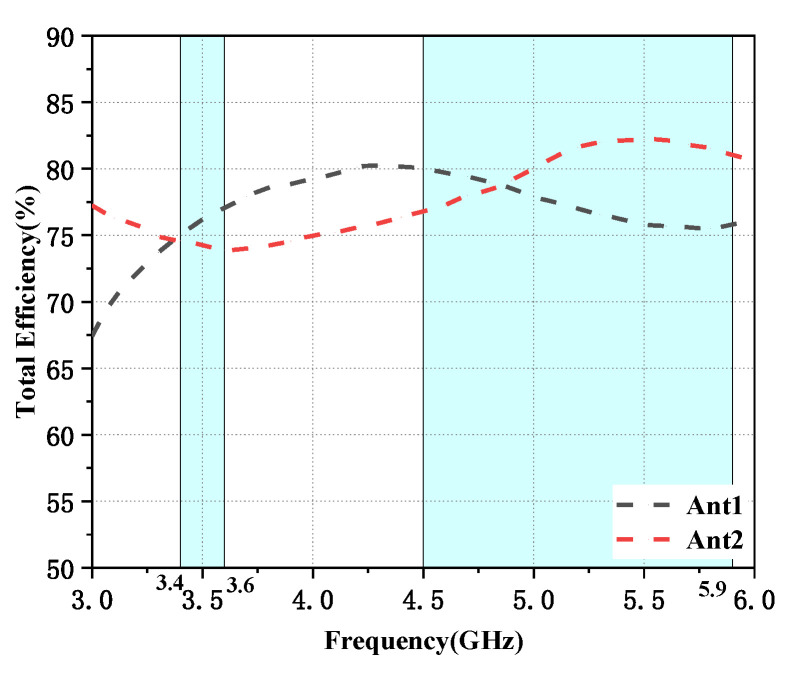
Variation of simulated values of antenna efficiency for different antenna elements.

**Figure 11:**
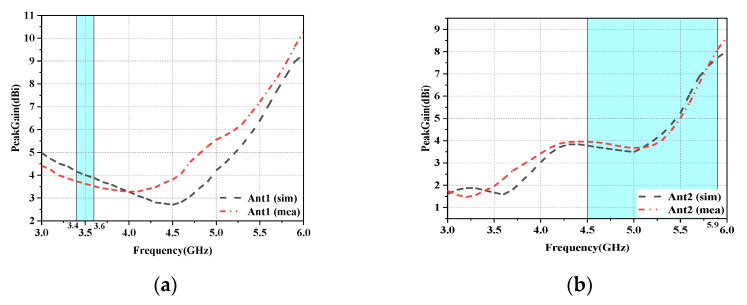
Antenna gain of the antenna element: (**a**) Ant1. (**b**) Ant2.

In this section, a preliminary analysis of the proposed twelve-cell antenna’s peak gain and overall efficiency findings is performed. The radiation efficiencies of the L-shaped antenna module and the folded monopole module (Ant1 and Ant2 as examples) are depicted in Figure 10. It is evident that the radiation efficiency of this antenna model is above 75% for both modules. Figure 11a,b depict the computed and observed peak gains of the L-shaped antenna element and the folded monopole element. The radiation gain of each antenna module is above 3.2 dBi in the covered frequency band. The increase in gain with increasing frequency is due to the increase in the effective size of the antenna at higher frequencies [30].

### 3.5. Radiation Patterns

**Figure 12:**
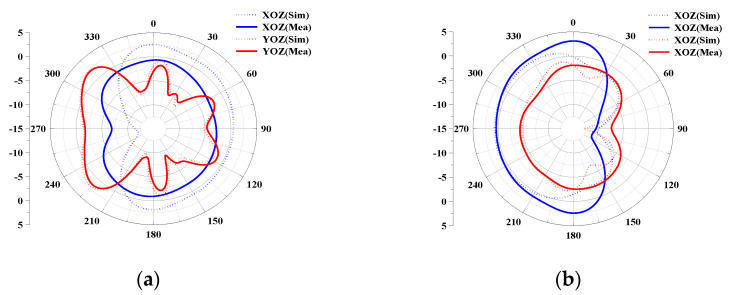
Simulated and measured 2D radiation maps of the proposed MIMO system: (**a**) Ant1 at 3.5 GHz. (**b**) Ant1 at 5 GHz.

In this subsection, the directional maps (E-plane and H-plane) of the proposed 12-port antenna array with L-shaped and folded monopole elements measured at two representative frequencies (3.5 GHz for the low-frequency band and 5.5 GHz for the high-frequency band) are discussed and analyzed separately, considering the similarity of the measured radiation, with the results of Ant1 and Ant2 as representative ones. Figure 12a shows that Ant1 radiates more efficiently between 210° and 330° in the YOZ plane at 3.5 GHz, and achieves essentially omnidirectional radiation in the XOZ plane. Similarly, at high frequencies, as shown in Figure 12b, Ant2 can achieve directional radiation from 180° to 360° at 5 GHz with a constant gain value, indicating that Ant2 has strong directional radiation performance.

### 3.6. User’s Hands Effects

**Figure 13:**
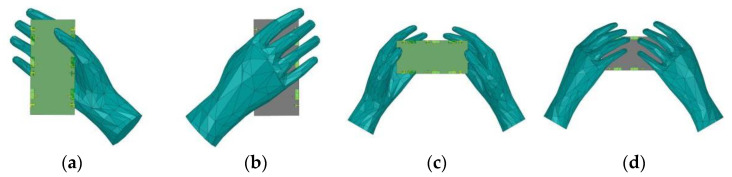
Different usage postures of SHM and DHM: (**a**) Front view of SHM. (**b**) Back view of SHM. (**c**) Front view of DHM. (**d**) Back view of DHM.

In this section, the effect of the user’s hand on the performance of the proposed antenna is investigated. There are two common usage modes of embedded smartphones, namely, talk mode (SHM) and data mode (DHM). The hand configurations of the proposed antenna design in two different modes of SHM and DHM are shown in Figure 13.

**Figure 14:**
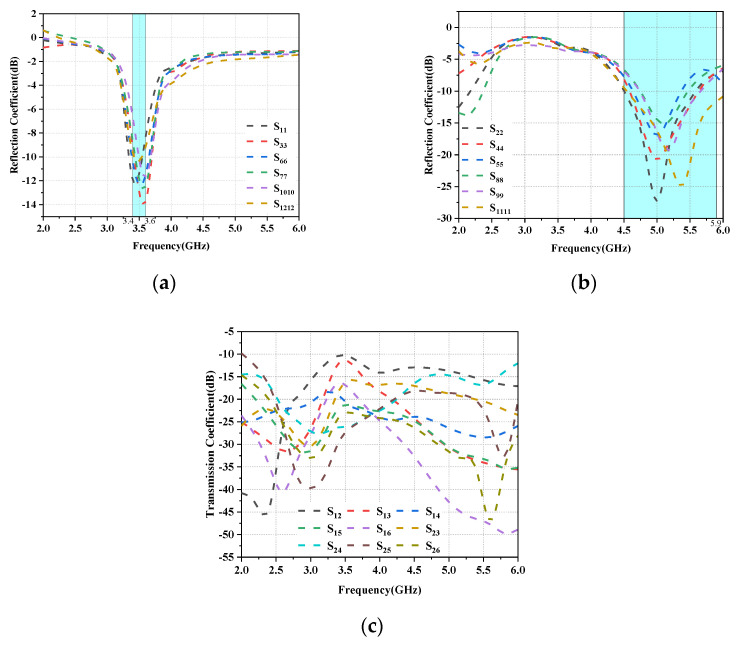
Simulated S-parameter results for SHM operation: (**a**) Reflection coefficient of L-shaped antenna module. (**b**) Reflection coefficient of the folded monopole module. (**c**) Transmission coefficient.

**Figure 15:**
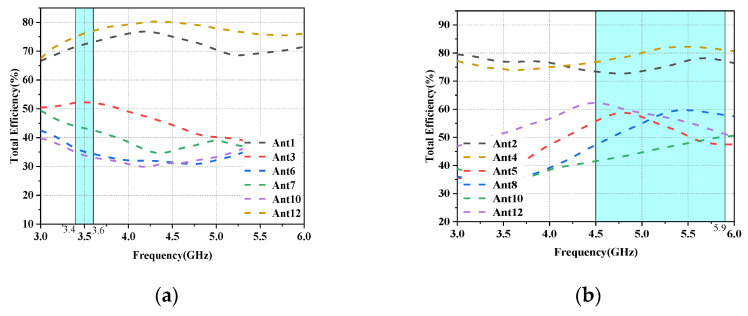
Antenna efficiency in SHM mode: (**a**) Efficiency of L-shaped antenna module. (**b**) Efficiency of the folded monopole module.

In the talk mode (SHM), the reflection coefficients of the L-shaped antenna and the folded monopole module are not significantly affected by the user’s hand, as shown in Figure 14a,b. However, the impedance matching of some antenna elements closer to the hand (Ant3, Ant6, Ant7, Ant10 and Ant11) is slightly shifted to a higher frequency band, but still covers the desired band. However, as shown in Figure 14c, their isolation is not affected too much, and all antennas still have isolation greater than 10 dB in the desired operating band. In addition, as shown in Figure 15a,b, due to the absorption of electromagnetic waves by the hand tissue, some elements of the radiation efficiency of the L-antenna module (Ant3, Ant6, Ant7 and Ant10) and some modules of the serpentine antenna (Ant5, Ant8, Ant10 and Ant12) drop to less than 60%, while other antennas still show greater than 75% efficiency due to being located far from the hand.

**Figure 16:**
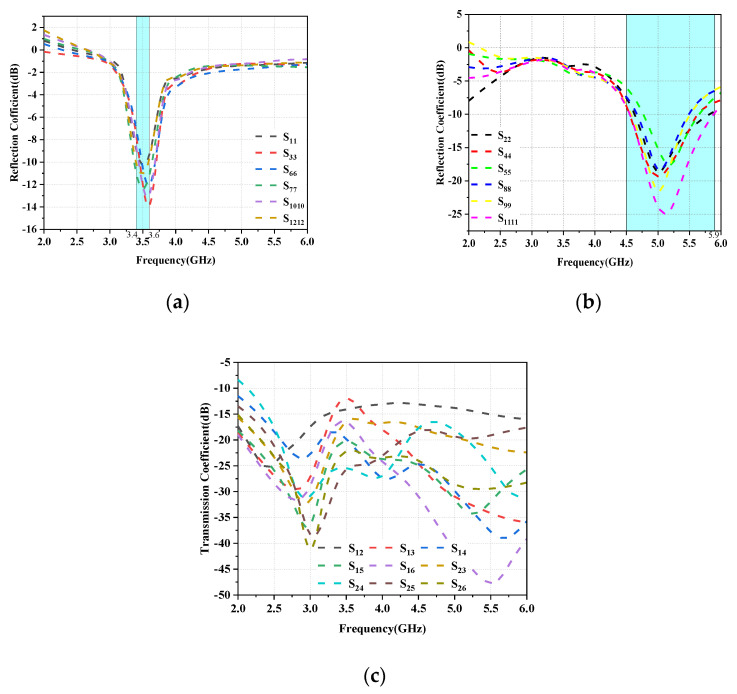
Simulated S-parameter results for DHM operation: (**a**) Reflection coefficient of L-shaped antenna module. (**b**) Reflection coefficient of the folded monopole module. (**c**) Transmission coefficient.

**Figure 17:**
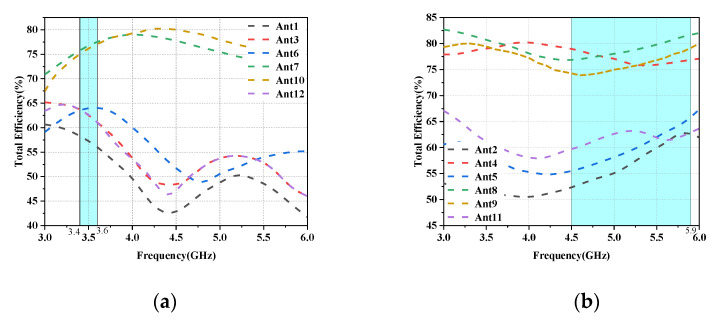
Antenna efficiency in DHM mode: (**a**) Efficiency of L-shaped antenna module. (**b**) Efficiency of the folded monopole module.

The functioning of the data mode (DHM) is depicted in the figure; as shown in Figure 16, the reflection coefficient and isolation are less impacted by the user’s hand, while the antenna efficiency is more affected as can be seen in Figure 17.

### 3.7. Performance Comparison

To highlight the advantages of the proposed antenna array, the comparison results of the same type of antennas are given in Table 2. It can be concluded from Table 2 that the proposed MIMO antenna has high isolation and a wider bandwidth to cover more communication bands compared to [31,32,33]; it has a lower ECC compared to [34,35]; and it has higher efficiency compared to [36,37]. After comparing with previous work, it can be seen that the MIMO antenna designed in this paper has higher antenna efficiency. The bandwidth, ECC and isolation have some advantages compared to previous work and can meet the communication requirements of 5G cell phones.

## 4. Conclusions

In this paper, a 12-port highly isolated broadband MIMO antenna system for 5G/WLAN applications is presented. The proposed MIMO antenna consists of an L-shaped antenna module covering the C-band (3.4–3.6 GHz) for 5G mobile applications and a folded monopole module covering the high band for 5G mobile applications as well as the WLAN band (4.5–5.9 GHz), where each antenna module consists of six identical antenna elements. In addition, the antenna pair composed of two antennas can achieve isolation higher than 11dB without adding additional decoupling facilities, obtains wideband characteristics, high isolation and low ECC, and also has good diversity performance, and therefore can be used in modern mobile terminals.

## Figures and Tables

**Table 1:** Dimensions of the single element.

Parameter	L1	L2	L3	L4	L5	L6	L7	L8	L9	L10
Value (mm)	2	3	8	3	2	7.5	5	10	5	4
Parameter	L_11_	L_12_	L_13_	W_1_	W_2_	W_3_	W_4_	W_5_	W_6_	W_7_
Value (mm)	5	6	17	0.5	1.5	2	1.5	1	0.6	6

**Table 2:** Performance comparison of various state-of-the-art 5G antennas.

References	Bandwidth (GHz)	Isolation (dB)	ECC	Total Efficiency (%)
[31]	3.4–3.6/4.8–5.1 (−6 dB)	>11.5	<0.08	>40
[32]	3.3–3.6/4.8–5.0 (−6 dB)	>10	<0.15	>60
[33]	3.3–5 (−6 dB)	>10	<0.3	>40.5
[34]	3.4–3.6 (−10 dB)	>12	<0.1	>50
[35]	3.4–3.6/5.15–5.93 (−6 dB)	>11.2	<0.08	>51
[36]	3.4–3.6 (−10 dB)	>10	<0.2	>62
[37]	2.496–2.69, 3.4–3.8 (−6 dB)	>10.5	<0.2	>44
Proposed	3.4–3.6 (−10 dB)/4.5–5.9 (−6 dB)	>11	<0.04	>75

## Data Availability

The simulated and measured data used to support the findings of this study are included within the article.
